# Lack of evidence for MHC-unrestricted (atypical) recognition of mucin by mucinous pancreatic tumour-reactive T-cells

**DOI:** 10.1054/bjoc.1999.0982

**Published:** 2000-01-18

**Authors:** R S Selvan, T N Pappas, F E Ward

**Affiliations:** Departments of Surgery and Immunology, Duke University, Box 3555, Medical Center, Durham, NC, 27710, USA

**Keywords:** mucin, tumour antigen, T-cell, immune response, pancreatic tumour

## Abstract

Cytotoxic T-cells generated against heterologous, mucinous pancreatic tumour cells were shown to recognize mucin in a major histocombatibility complex (MHC)-unrestricted fashion. In contrast, the present study demonstrates a typical allogeneic response of heterologous cytotoxic T-cells established against mucin-expressing pancreatic tumour cells. Heterologous cytotoxic T cells lysed targets that were used as stimulators and other targets that shared human leucocyte antigen (HLA) with the stimulator. These cytotoxic T-cells lysed mucin-expressing stimulator cells but not autologous tumour cells in spite of expressing mucin on their surface. Likewise, tumour-infiltrating CD4^+^T-cells proliferated against its own tumour cell target, while such T-cells did not respond to heterologous, mucin-expressing pancreatic tumour cells. Culturing heterologous tumour-specific cytotoxic T-cells with purified pancreatic tumour cell-mucin rendered them unresponsive to their target cells. Furthermore, purified mucin did not produce a mucin-specific response in mucinous pancreatic tumour patients' primary T-cells even in the presence of antigen-presenting cells. Our study finds no evidence for MHC-unrestricted recognition of mucin by pancreatic cancer patients' T-cells. © 2000 Cancer Research Campaign


British Journal of Cancer (2000) **82**(3), 691–701
© 2000 Cancer Research Campaign
DOI: 10.1054/ bjoc.1999.0982, available online at http://www.idealibrary.com on IDEAL®

### Lack of evidence for MHC-unrestricted (atypical) recognition of mucin by mucinous pancreatic tumour-reactive T-cells

**RS Selvan[1], TN Pappas[1] and FE Ward[2]**

Departments of [1]Surgery and [2]Immunology, Duke University, Box 3555, Medical Center, Durham, NC 27710, USA

**Summary** Cytotoxic T-cells generated against heterologous, mucinous pancreatic tumour cells were shown to recognize mucin in a major histocombatibility complex (MHC)-unrestricted fashion. In contrast, the present study demonstrates a typical allogeneic response of heterologous cytotoxic T-cells established against mucin-expressing pancreatic tumour cells. Heterologous cytotoxic T cells lysed targets that were used as stimulators and other targets that shared human leucocyte antigen (HLA) with the stimulator. These cytotoxic T-cells lysed mucin-expressing stimulator cells but not autologous tumour cells in spite of expressing mucin on their surface. Likewise, tumour-infiltrating CD4$^+$ T-cells proliferated against its own tumour cell target, while such T-cells did not respond to heterologous, mucin-expressing pancreatic tumour cells. Culturing heterologous tumour-specific cytotoxic T-cells with purified pancreatic tumour cell-mucin rendered them unresponsive to their target cells. Furthermore, purified mucin did not produce a mucin-specific response in mucinous pancreatic tumour patients' primary T-cells even in the presence of antigen-presenting cells. Our study finds no evidence for MHC-unrestricted recognition of mucin by pancreatic cancer patients' T-cells. © 2000 Cancer Research Campaign

**Keywords:** mucin; tumour antigen; T-cell; immune response; pancreatic tumour

Mucin (MUC-1) was initially identified with DU-PAN-2 murine monoclonal antibody raised against a pancreatic adenocarcinoma cell line, HPAF (Lan et al, 1985). Mucin detected on the surface of established pancreatic tumour cells was also abundantly expressed on tumour tissues (Borowitz et al, 1984). MUC-1 has also been shown to be recognized by T-cells in vitro. Barnd et al (1989) demonstrated that heterologous cytotoxic T-cells (CTLs) generated from lymph node cells of a patient recognized tumour-associated mucin in a specific, major histocombatibility complex (MHC)-unrestricted fashion. Since a pancreatic tumour cell-reactive autologous T-cell system was not available, heterologous pancreatic tumour cell lines were used as stimulators of lymph node cells (Barnd et al, 1989). MHC-unrestricted CTLs generated against heterologous, mucin-expressing pancreatic tumour cells were also reported to lyse breast tumour cells which expressed MUC-1 (Barnd et al, 1989; Jerome et al, 1991). It was postulated that tandem repeats and aberrant glycosylation of tumour cell mucin were responsible for such a MHC-unrestricted recognition by heterologous T-cells (Barnd et al, 1989; Jerome et al, 1991; Poland et al, 1997). Recently, Margarian-Blander et al (1998) demonstrated that direct recognition of the MUC-1 peptide epitope by the T-cell receptor (TCR) in the absence of presentation by the MHC induced a partial signal. However, mucin has yet to be demonstrated as an antigen recognized by pancreatic tumour cell-reactive, autologous T-cells. In this study, we investigated whether pancreatic tumour-associated mucin is recognized by pancreatic tumour-patients' T-cells in a MHC-unrestricted fashion using human leucocyte antigen (HLA)-defined heterologous and autologous systems, and whether purified mucin could elicit tumour-reactive T-cells. Our study does not support the claim that mucinous pancreatic tumour cell-reactive T-cells recognize mucin in MHC-unrestricted fashion.

*Received 2 June 1999*
*Revised 2 September 1999*
*Accepted 6 September 1999*

*Correspondence to:* RS Selvan, Senior Scientist, Hoag Cancer Center, Building 41, Suite 3F, 1 Hoag Drive, Newport Beach, CA 92663, USA

### MATERIALS AND METHODS

### Tumour cell lines

Previously established pancreatic adenocarcinoma cell lines HPAF, CAPAN-1, CAPAN-2, T3M4, COLO-357 and PANC-1, melanoma cell line SKMEL-14, breast carcinoma cell line SKBR-3 were cultured in complete minimal essential medium (MEM), containing with 100 U ml$^{-1}$ penicillin, 100 µg ml$^{-1}$ streptomycin, 2 mM glutamine, and 10% heat-inactivated fetal bovine serum (FBS; Barnd et al, 1989; Wahab and Metzgar, 1991). Myelogenous leukemia cell line K-562 (obtained from Dr Zeinab Wahab, Duke University) was cultured in complete RPMI-1640 medium. The adherent tumour cell cultures were transferred regularly by trypsinization (0.25% trypsin with 0.2% EDTA). Tissue culture reagents were obtained from Gibco-BRL (Grand Island, NY, USA). Cell lines were found to contain no mycoplasm determined by enzyme-linked immunosorbent assay (ELISA) kit obtained from Boehringer Mannheim (Indianapolis, IN, USA).

### Establishment of primary pancreatic tumour cell line

WM-tumour explant cultures were established from a moderately differentiated primary pancreatic tumour mass of a patient who underwent Whipple procedure, using standard techniques (Tan et al, 1986). Briefly, a tissue sample was washed and teased in a tissue culture dish (Falcon, Becton Dickinson, Franklin Lakes, NJ, USA) in a medium consisting of 50% (v/v) Dulbecco's modified Eagle's medium (DMEM), 50% (v/v) F-12 Ham medium supplemented with 10% FBS, 5 μg $ml^{-1}$ insulin, 5 μg $ml^{-1}$ transferrin and 5 ng $ml^{-1}$ selenium, 100 U $ml^{-1}$ penicillin and 100 μg $ml^{-1}$ streptomycin, 2 mM glutamine and $5 \times 10^{-5}$ M 2-mercaptoethanol. Small tissue fragments and freed cells were separated from the large fragments and transferred into a 50 ml centrifuge tube (Corning Inc., Corning, NY, USA). Large fragments (mostly connective tissues) remaining in tissue culture dish were discarded. The suspension containing small tissue fragments and freed cells was allowed to stand at room temperature for 5 min. After the small tissue fragments had settled, the cell suspension was transferred to another 50 ml centrifuge tube. The remaining small tissue fragments and freed cells were washed once by subjecting to centrifugation at 1000 rpm for 10 min. Both preparations were resuspended in complete DMEM/F-12 Ham medium and placed in 25-$cm^2$ tissue culture flasks (Costar, Cambridge, MA, USA) at 37°C in humidified atmosphere containing 5% carbon dioxide. Within 2–3 weeks, islands of epithelial cells were allowed to grow by preventing the outgrowth of fibroblasts using differential trypsinization procedure with low trypsin (0.05% trypsin and 0.02% EDTA; Gibco-BRL). By periodically repeating this procedure, primary pancreatic tumour cell line, WM, was established.

### Flow cytometry and immunocytochemistry of tumour cells

The cultured WM and HPAF tumour cells were first trypsinized and immediately resuspended in FBS containing medium. After being washed twice, cells were resuspended in complete medium and allowed to recover antigens by incubation at room temperature for 1–2 h. The expression of MHC-class I and -class II molecules on the surface of tumour cells was determined using indirect immunofluoresence assay essentially as described by us (Selvan et al, 1991). The tumour cells were stained with anti-HLA class I (W6/32) and anti-HLA class II (L243) antibodies (affinity purified antibodies from tissue culture supernatant of hybridomas were obtained from ATCC, Rockville, MD, USA) respectively. The control cells were stained with isotype-matched antibody ($IgG_{2a}$; Sigma, St Louis, MO, USA). All samples were stained with fluorescein-conjugated goat anti-mouse antibodies (Organon Teknika, Durham, NC, USA) and analysed for fluorescence intensity in an Ortho Cytofluorograph 50-H (Ortho Instruments, Westwood, MA, USA). The tumour cells were further assessed for the expression of mucin using immunocytochemical procedure as described elsewhere (Borowitz et al, 1984). Briefly, tumour cells were grown to confluence in multi-chamber culture slides (Lab-Tek, NUNC, Inc., Laperville, IL, USA). Cells were washed with phosphate-buffered saline (PBS) and fixed with cold acetone (–20°C). The fixed cells were blocked with PBS containing 1% bovine serum albumin (BSA; Sigma) and incubated with primary antibody DU-PAN-2 (IgM; hybridoma culture supernatant was a gift from Dr Zeinab Wahab, Duke University) or SP-1 supernatant. After washing, the cells were incubated with goat anti-mouse IgG peroxidase-conjugated antibody. The cells were washed in PBS, stained with diaminobenzidine and counter stained with Gill's haematoxylin.

### Immortalization of B-cells

Peripheral blood B lymphocytes from pancreatic tumour patients were immortalized with tissue culture fluid containing Epstein–Barr virus (EBV) from the marmoset cell line B95-8 essentially as described elsewhere (Miller and Lipman, 1973). Briefly, $5 \times 10^6$ peripheral blood lymphocytes (PBLs) were infected with EBV in the presence of 20 μg $ml^{-1}$ cyclosporine and cultured in RPMI-1640 medium containing 10% FBS for 4–6 weeks during which time the immortalized B-cells exhibited active growth.

### HLA typing

HLA typing of tumour cells and PBLs was carried out using complement-dependent microcytotoxicity assay with anti-HLA monoclonal and polyclonal antibodies (Pollack et al, 1981).

### Tumour-specific T-cell lines

Tumour cell-specific heterologous T-cell lines derived from draining lymph nodes of pancreatic adenocarcinoma patients, were established by stimulation with irradiated (6000 rad) heterologous pancreatic tumour cells (HPAF, T3M4 and PANC-1) and 5 U $ml^{-1}$ human recombinant interleukin-2 (IL-2; DuPont, Wilmington, DE, USA) basically as described by Barnd et al (1989). Tumour-infiltrating lymphocytes (TILs), and tumour cell-reactive autologous T-cells from peripheral blood mononuclear cells (PBMCs) were established with irradiated autologous tumour cells WM and IL-2 as described by us and others (Slovin et al, 1986; Belldegrun et al, 1989; Selvan et al, 1991). Leucocyte lineage-specific antigens on the tumour-specific T-cells were determined using immunofluorescence followed by cytofluorometric analysis using anti-CD3, anti-CD4, anti-CD8, anti-CD19, anti-CD20, anti-CD16 and CD56 antibodies (tissue culture supernatants of hybridomas obtained from ATCC).

### Lymphokine-activated killer (LAK) cells

Effector lymphokine-activated killer cells were generated by culturing PBMCs from mucinous pancreatic adenocarcinoma patients with 1000 U $ml^{-1}$ of IL-2 for 5 days (Grimm et al, 1982).

### Culturing of T cells with purified mucin

Purified mucin used in this investigation was provided by Dr MS Lan, a coinvestigator of the report by Barnd et al (1989) and the lot of mucin preparation is the same as that used by Barnd et al (1989) (M Lan, personal communication). Mucin was prepared as described elsewhere (Lan et al, 1985, 1987). The quantity and purity of mucin preparations were qualified by the levels of reactivity against DU-PAN-2 antibody using competitive inhibition radioimmunoassay (RIA; Metzgar et al, 1984) and Western blot analysis respectively (Lan et al, 1985, 1987). Rather than express the results as percent inhibition and titre, the quantity of DU-PAN-2 antigen in a sample was expressed as arbitrary units $ml^{-1}$ based on reference to the partially purified standard antigen sample. The amount of DU-PAN-2 antigen in 20 μl of a 1:500 dilution of the standard antigen preparation was designated as 100 units $ml^{-1}$ (Metzgar et al, 1984). The heterogeneity of mucin antigen migration on the gel indicated that the monoclonal antibody DU-PAN-2

 © 2000 Cancer Research Campaign recognized multiple mucin molecules which bore the same epitope (Lan et al, 1985, 1987). The buoyant densities and amino acid compositions of purified fractions (I and II) suggested that DU-PAN-2 antibody-reacting antigen was mucin-like glycoprotein (Lan et al, 1987). IL-2 used in our experiments was obtained from Dupont (Wilmington, DE, USA) which is the same as used by Barnd et al (1989). One times $10^6$ heterologous tumour-specific T-cell lines per ml (from lymph node cells of a mucinous pancreatic adenocarcinoma patient, established against mucinous T3M4 or non-mucinous PANC-1 pancreatic tumour cell lines) were repeatedly cultured with 1900 U $ml^{-1}$ mucin and 5 U $ml^{-1}$ IL-2 in 24-well flat-bottomed tissue culture plates (Costar, Costar Corp., Cambridge, MA, USA). Similarly, freshly isolated PBMCs from pancreatic adenocarcinoma patients were cultured with mucin plus IL-2. The leucocyte cultures were periodically cleared off excess debris by centrifugation through Ficoll gradient (Organon Technica). The cultures were repeatedly restimulated with mucin after a cycle of culturing with mucin plus IL-2 for 3–4 days followed by IL-2 alone for 2–3 days. Some of the cultured cells were tested in triplicates for their ability to lyse T3M4, HPAF, PANC-1 and natural killer (NK) cell target K-562 or to proliferate against purified mucin. To determine the proliferation, $10^5$ PBMCs from cancer patients were incubated in triplicates with 5 U $ml^{-1}$ IL-2 or varying concentrations (475 U $ml^{-1}$ to 2375 U $ml^{-1}$) of mucin in the presence or absence of 5 U $ml^{-1}$ IL-2 in 96-well flat-bottomed tissue culture plates (Costar). After 72 h, the wells were pulsed with 1 μCi per well [$^3$H]thymidine and incorporation of [$^3$H]thymidine into DNA of proliferating cells was measured as described below.

### Cytolytic assay

The cytolytic activity of tumour-infiltrating lymphocytes, peripheral blood tumour-specific T-cells, LAK cells and mucin-stimulated T-cells or PBMCs was assayed using 4 h Chromium-51 release assay (Barnd et al, 1989; Selvan et al, 1990, 1991). As targets, we used several well established tumour cell lines including the heterologous pancreatic cancer cell lines HPAF, T3M4, PANC-1, COLO-357, CAPAN-1 and CAPAN-2, the autologous pancreatic cancer cell line WM and EBV-immortalized B cells, the breast tumour cell line SKBR-3, the melanoma cell line SKMEL-14 and the myelogenous leukaemia cell line K-562 (Barnd et al, 1989). In some cases, target cells were preincubated with 20 μg $ml^{-1}$ control antibody ($IgG_{2a}$; Sigma) or anti-MHC class I antibody (W6/32) to block MHC class I-mediated cytolytic response. These target cells were then washed three times in RPMI medium and the cytotoxicity assay in triplicate samples was performed at various effector to target ratios as described above.

### Proliferation assay

The proliferative rate was determined in triplicate samples for isolated tumour-infiltrating T-cells and peripheral blood tumour-reactive T-cells (Barnd et al, 1989; Selvan et al, 1990, 1991). Briefly, $10^5$ responder resting T-cells were incubated with $10^4$ irradiated autologous or heterologous stimulator cells for 3 days at 37°C in complete medium supplemented with 10% FBS in the presence or absence of 1 U $ml^{-1}$ IL-2. The cells were then pulse-labelled with 1 μCi per well of [$^3$H]thymidine for the last 18 h at 37°C and harvested using a Skatron cell harvester (Skatron Instruments Inc., Sterling, VA, USA). The incorporation of [$^3$H]thymidine was quantitated using a liquid scintillation counter (Wallac 1409, Wallac Oy, Turku, Finland). In some cases, irradiated stimulator cells were preincubated with 20 μg $ml^{-1}$ control antibody ($IgG_{2a}$; Sigma) or anti-MHC class II antibody (L243) to block MHC class II-mediated proliferative response. In other cases, 10 μg $ml^{-1}$ affinity purified antibody against pancreatic tumour mucin, DU-PAN-2 or SP-1 supernatant was included (Barnd et al, 1989). These cells were then washed three times in RPMI medium and added at proper concentration to the responder T-cells. The proliferative response of T-cells was measured as described above.

### Statistical analysis

All determinations were made in triplicate and data are reported as the mean ± s.e.m. The statistical analysis of data was carried out using Student's *t*-test, and $P < 0.05$ between two groups were considered to be statistically significant.

### RESULTS

### Heterologous pancreatic tumour-reactive cytotoxic T-cells do not recognize pancreatic tumour cells in the context of MHC-unrestricted mucin

In an effort to determine whether heterologous cytotoxic T cells recognize mucin expressed by human pancreatic tumour cells, cytotoxic T-cell lines (TP and RM) were established from draining lymph nodes of pancreatic tumour patients by stimulating with mucin-expressing (T3M4 or HPAF) and non-mucin-expressing (PANC-1) tumour cells (Table 1). The tumour patients had very high levels of mucin in the serum at the time of surgery, determined by enzyme-linked immunosorbent assay (ELISA) using DU-PAN-2 monoclonal antibody (Metzgar et al, 1984; data not shown). Cytotoxic T-cells derived from draining lymph node cells of a pancreatic adenocarcinoma patient (TP) were generated against heterologous, mucin-expressing pancreatic tumour cell line T3M4 in the presence of 5 U $ml^{-1}$ IL-2. The established cytotoxic T-cells effectively lysed the stimulator cells, T3M4 (78% lysis at effector:target ratio of 25:1) as well as another target (CAPAN-1) that expressed mucin (87% lysis). The same effector cells, however, did not lyse other mucin-expressing pancreatic tumour cell lines such as HPAF or COLO-357 but exhibited a low level reactivity (20–23% lysis) towards mucin-expressing CAPAN-2 cells as well as non-mucin-expressing PANC-1 cells. In a similar fashion, TP cytotoxic T-cells established against mucin-expressing HPAF cells effectively lysed the HPAF cells (71% lysis). These cytotoxic T-cells elicited a range of low level reactivity (19–28% lysis) towards mucin-expressing CAPAN-1 and T3M4 cells, and non-mucin-expressing PANC-1 cells. TP cytotoxic T-cells established against non-mucin-expressing PANC-1 cells very effectively lysed PANC-1 cells (78% lysis) and melanoma cell line SKMEL-14 (76% lysis) and, at a low level (23% lysis) mucin-expressing T3M4 cells but not other mucin-expressing pancreatic tumour cells. A similar reactivity was also seen with RM cytotoxic cells established from pancreatic adenocarcinoma patient against T3M4, HPAF or PANC-1 cells (Table 1). Interestingly, none of the heterologous T-cells established against mucin-expressing pancreatic tumour cells were able to lyse mucin-expressing breast tumour cell line SKBR-3. Previous studies from elsewhere have shown that heterologous, pancreatic

*© 2000 Cancer Research Campaign* 

**Table 1** Tumour target cell lysis by heterologous pancreatic tumour-reactive T-cells[a]

| Responder T-cells | Targets tested | % Specific lysis by cytotoxic T-cells established against heterologous tumour cells[b] T3M4 | HPAF | PANC-1 |
|---|---|---|---|---|
| TP | Pancreatic adenocarcinoma | | | |
| | CAPAN-1 | 86.88 ± 1.57 | 21.46 ± 1.00 | 1.11 ± 0.61 |
| | CAPAN-2 | 22.75 ± 1.19 | –3.71 ± 0.31 | 2.44 ± 0.57 |
| | T3M4 | 78.17 ± 0.08 | 27.93 ± 0.75 | 23.37 ± 0.31 |
| | HPAF | 2.30 ± 0.57 | 70.60 ± 0.58 | 2.83 ± 0.14 |
| | COLO-357 | 1.87 ± 0.62 | 4.71 ± 0.28 | 2.52 ± 0.99 |
| | PANC-1 (non-mucinous) | 20.43 ± 0.91 | 18.60 ± 0.51 | 78.20 ± 0.86 |
| | Melanoma | | | |
| | SKMEL-14 | 11.20 ± 0.35 | 9.02 ± 0.28 | 75.70 ± 0.80 |
| | Myelogenous leukaemia | | | |
| | K-562 | 4.91 ± 0.84 | 4.94 ± 0.56 | 2.99 ± 0.73 |
| RM | Pancreatic adenocarcinoma | | | |
| | CAPAN-1 | 65.02 ± 1.85 | 26.19 ± 2.17 | 7.81 ± 0.42 |
| | CAPAN-2 | 35.86 ± 2.09 | 24.91 ± 2.64 | 1.31 ± 0.83 |
| | T3M4 | 73.00 ± 3.45 | 28.25 ± 1.04 | 11.99 ± 0.84 |
| | HPAF | NT | 33.05 ± 1.47 | NT |
| | COLO-357 | 29.31 ± 0.86 | –5.83 ± 2.30 | 3.50 ± 1.26 |
| | PANC-1 (non-mucinous) | 22.51 ± 0.71 | 38.59 ± 2.69 | 61.84 ± 2.95 |
| | Melanoma: | | | |
| | SKMEL-14 | 1.97 ± 0.28 | 9.17 ± 0.26 | 22.17 ± 0.57 |
| | Breast carcinoma: | | | |
| | SKBR-3 | –0.25 ± 0.41 | 1.38 ± 0.93 | –0.31 ± 0.64 |
| | Myelogenous leukaemia | | | |
| | K-562 | 11.60 ± 0.28 | NT | 9.51 ± 1.06 |

[a]Cytotoxic T-cells TP and RM were established from tumour-draining lymph nodes of pancreatic adenocarcinoma patients against heterologous pancreatic tumour cells T3M4, HPAF or PANC-1 by repeated stimulation with irradiated tumour cells in the presence of 5 U ml$^{-1}$ interleukin-2. The data represent the cytolytic activity of T-cell lines assessed at about 2 months of continuous culture against Chromium-51-labelled tumour cell targets using various effector (E) to target (T) ratios. [b]Values represent percentage specific lysis obtained at E:T 25:1 in a 4 h Chromium-51 release assay. NT, not tested.

**Table 2** Heterologous pancreatic tumour-reactive cytotoxic T cells do not recognize pancreatic tumour cells in the context of MHC-unrestricted mucin

| Effector | HLA-typing of effector cells (Class I) | Target | HLA-typing of target cells (Class I) | % Cytotoxicity[d] |
|---|---|---|---|---|
| 1. RC-T cell line against WM-tumour cells[a] | A3,A30; B18,B27; Bw4,Bw6 | WM | **A1***, A3#; B7,B37; Bw4,Bw6; Cw6 | 35.21 ± 1.79 |
| | | HPAF | **A1**,A34; B8,B22; Bw6; Cw3 | 55.58 ± 1.03 |
| | | K-562 | HLA Class I-negative (NK Cell Target) | 0.01 ± 0.80 |
| 2. WM-T cell line against HPAF-tumour cells[b] | A1,A3; B7,B37; Bw4,Bw6; Cw6 | HPAF | A1,A34; **B8**,B22; Bw6; Cw3 | 39.83 ± 1.71 |
| | | WM | A1,A3; B7,B37; Bw4,Bw6; Cw6 | 1.27 ± 0.53 |
| | | FW-PBL Blasts[c] | A1; **B8**; Bw6; Cw7 (homozygous) | 61.27 ± 1.61 |
| | | K-562 | HLA Class I-negative (NK Cell Target) | –1.61 ± 0.64 |

[a]RC- and [b]WM-cytolytic effector T-cell lines were established from peripheral blood mononuclear cells of pancreatic adenocarcinoma patients against heterologous, mucin-expressing pancreatic tumour cells, WM and HPAF respectively, in the presence of 5 U ml$^{-1}$ interleukin-2. [c]FW-peripheral blood cells were stimulated with PHA (2 μg ml$^{-1}$) for 48 h and the resultant blast cells were labelled with Chromium-51 and used as target cells. [d]Cytolytic activity of T-cells against indicated tumour targets were assayed at about 3 months after continuous culture using various effector (E) to target (T) ratios. The values represent percentage specific lysis obtained at E:T 25:1 in a 4 h Chromium-51 release assay. The results shown are representative of five experiments. *Bold face denotes shared-MHC class I alleles on target cells recognized by effector cytotoxic T-cells. [#]Underlining denotes matched-MHC class I alleles between responder and stimulator or target cells.

*© 2000 Cancer Research Campaign*

**Table 3** Anti-MHC class I antibody inhibits heterologous, pancreatic tumour-reactive CTL killing of mucin-expressing and -non-expressing target cells

| Effector | Target | % Cytotoxicity[d] |
|---|---|---|
| 1. RC-T cell line against WM-tumour cells[a] | WM + Control antibody | 37.83 ± 0.80 |
| | WM + W6/32 antibody | 5.33 ± 0.48 (86%) |
| | HPAF + Control antibody | 49.23 ± 2.91 |
| | HPAF + W6/32 antibody | 8.14 ± 1.83 (83%) |
| 2. WM-T cell line against HPAF-tumour cells[b] | HPAF + Control antibody | 42.45 ± 1.00 |
| | HPAF + W6/32 antibody | 7.32 ± 1.63 (83%) |
| | WM + Control antibody | 1.37 ± 0.50 |
| | WM + W6/32 antibody | 1.41 ± 0.55 |
| | FW-PBL Blasts[c] + Control antibody | 57.23 ± 2.69 |
| | FW-PBL Blasts + W6/32 antibody | 9.44 ± 1.37 (84%) |

[a]RC- and [b]WM-cytolytic effector T-cell lines were established from peripheral blood mononuclear cells of pancreatic adenocarcinoma patients against heterologous, mucin-expressing pancreatic tumour cells, WM and HPAF respectively, in the presence of 5 U $ml^{-1}$ interleukin-2. [c]FW-peripheral blood cells were stimulated with PHA (2 $\mu g\ ml^{-1}$) for 48 h and the resultant blast cells were labelled with Chromium-51 and used as target cells. [d]Cytolytic activity of T-cells against indicated tumour targets preincubated (60 min at 37°C) with 20 $\mu g\ ml^{-1}$ control antibody ($IgG_{2a}$) or W6/32 antibody and washed in RPMI medium, were used in cytotoxic assay employing various effector (E) to target (T) ratios. The values represent percentage specific lysis obtained at E:T 25:1 in a 4 h Chromium-51 release assay. HLA pattern of effector and target cells are given in Table 2. Similar results were obtained in another experiment. The values in parentheses represent percent inhibition of cytotoxicity over respective controls.

tumour cell-reactive T-cells also recognized breast tumour cells due to the expression of similar mucin (Barnd et al, 1989; Jerome et al, 1991). The present results show that cytotoxic T-cells derived from patients with mucin-expressing pancreatic adenocarcinoma exhibit reactivity in a typical fashion by effectively lysing predominantly the cells that were used as stimulators. A cytolytic response seen against few other targets in this study suggests that these target cells have HLA-alleles matched with the stimulators.

To more precisely understand the reactivity of heterologous cytotoxic T-cells towards mucin-expressing pancreatic tumour cells, a set of HLA-defined tumour patient cytotoxic T-cells were established by stimulation with HLA-defined heterologous, mucin-expressing pancreatic cancer cells in the presence of IL-2. As shown in Table 2, cytotoxic T-cell responses against heterologous, mucin-expressing pancreatic tumour cell targets occurred primarily as a typical allogeneic response. RC cytotoxic T-cells lysed the heterologous target tumour cells, WM, that were used as stimulators (35% lysis at E:T 25:1) as well as the target HPAF cells that had a matching MHC class I allele (A1) (56% lysis at E:T 25:1). In a similar fashion, WM cytotoxic T-cells established against HPAF cells, lysed HPAF cells (40% lysis at E:T 25:1) as well as non-mucinous target PBL-blast cells (61% lysis at E:T 25:1) that had a MHC-class I allele (B8) matched to HPAF cells. These CTLs (WM), however, did not lyse autologous tumour cells in spite of expressing mucin on their surface. Preincubation of target cells with W6/32 antibody blocked the cytotoxicity (83–86% inhibition) of both RC as well as WM CTLs irrespective of whether target cells expressed mucin (Table 3). The results demonstrate that heterologous T-cells generated against mucin-expressing pancreatic tumour cells appear to lyse targets, not in the context of mucin but in a typical alloreaction.

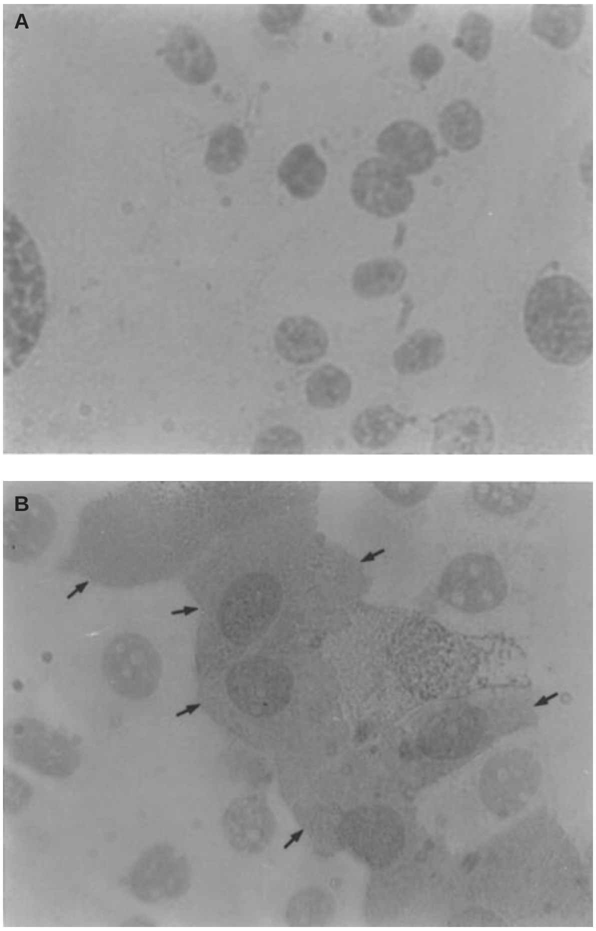

**Figure 1** Expression of mucin on WM-pancreatic tumour cells. Cultured WM cells grown to confluence on a multichamber slide (NUNC) were stained with (**A**) control antibody (SP-1 supernatant), and (**B**) anti-mucin IgM antibody (DU-PAN-2) followed by peroxidase-conjugated goat anti-mouse antibody and developed with the substrate diaminobenzidine (Borowitz et al, 1984). Immunostained cells were counter stained with Gill's haematoxylin

### Autologous pancreatic tumour-reactive T-cells do not recognize mucin on tumour cells in MHC-unrestricted fashion

To circumvent the response of heterologous T-cells to disparate MHC molecules on heterologous pancreatic tumour cells, an autologous pancreatic tumour cell-reactive T-cell system (WM) was established from a surgically excised primary pancreatic adenocarcinoma. The system comprised of a tumour cell line, tumour cell-reactive T-cell lines from TILs and peripheral blood lymphocytes, and EBV-transformed B-cells. Characterization of the tumour cell line revealed that tumour cells expressed mucin as determined by immunoperoxidase staining with DU-PAN-2 antibody (Figure 1) (Borowitz et al, 1984), and that the tumour cell line was MHC class I as well as class II-positive (Figure 2). Both TILs and tumour cell-reactive T-cells from peripheral blood proliferated against autologous tumour cells, WM, but not against heterologous pancreatic tumour cells, HPAF (Table 4), and were shown to be $CD4^+$ T-cells (data not shown). These cells exhibited no cytolytic function against the autologous tumour cells, WM (data not shown). Since the TIL line was established in the pres-

© 2000 Cancer Research Campaign

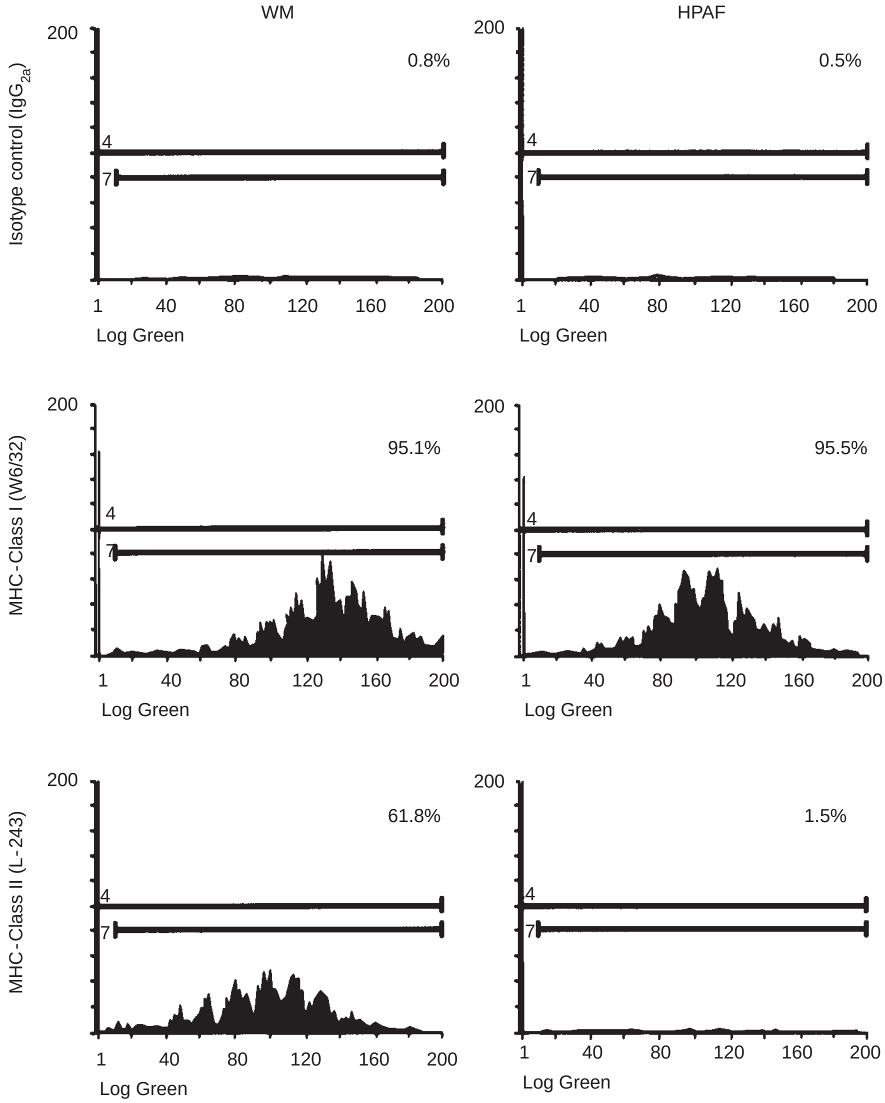

**Figure 2** Expression of MHC-Class I and Class II molecules on pancreatic tumour cell lines. The cultured tumour cells (WM and HPAF) were stained with isotype control antibody ($IgG_{2a}$), and antibodies against MHC-Class I (W6/32) and MHC-Class II (L243) followed by fluorescein-conjugated goat anti-mouse antibodies for flow cytometry analysis ence of IL-2, these cells expressed a proliferative response to IL-2 alone. When irradiated autologous tumour cells were added with IL-2, a tumour-specific proliferative response was consistently observed ($P < 0.05$). Such a proliferative response of these T-cells could not be detected against heterologous, mucin-expressing pancreatic tumour cells, HPAF (Table 4), or autologous EBV-immortalized B-cells (Table 5). In addition, inclusion of anti-MHC class II antibody (L243) but not anti-mucin antibody (DU-PAN-2) inhibited the proliferative response of TILs against autologous mucin-expressing pancreatic tumour cells, WM ($P < 0.05$; Table 5). Autologous tumour-specific T-cell lines established from PBLs of the same patient elicited a significant response similar to tumour-infiltrating lymphocytes ($P < 0.01$; Table 4). The results do not support the contention that MHC-unrestricted mucin is involved in the patients' T cell-recognition of pancreatic tumour cells.

### Lymphokine-activated killer cells do not recognize pancreatic tumour cells in the context of mucin

We investigated whether HLA-defined autologous, and heterologous, lymphokine-activated killer cells (WM- and RC-LAK cells respectively) recognize pancreatic tumour-associated mucin (Table 6). LAK cells, derived from PBMCs, lysed heterologous, mucin-expressing pancreatic tumour cells, HPAF (50% lysis at E:T 25:1), but not the autologous, mucin-expressing pancreatic tumour cells, WM (2% lysis at E:T 25:1). It is not known whether the non-reactivity of autologous LAK cells to its target is a specific response to the present system or a general phenomenon. Heterologous LAK cells (RC-LAK cells) established from peripheral blood mononuclear cells of a pancreatic adenocarcinoma patient, on the other hand, lysed WM cells (27% at E:T 25:1) as well as HPAF (54% lysis at E:T 25:1) tumour cells. As

© 2000 Cancer Research Campaign

**Table 4** Autologous but not heterologous mucinous pancreatic tumour cells enhance the stimulatory effect of IL-2 on tumour-infiltrating T-cell line and tumour-specific peripheral blood T-cell line[a]

| Responder | Stimulator | Proliferative Index Expt. 1 | Expt. 2 | Expt. 3 |
|---|---|---|---|---|
| A. | | | | |
| 1. WM-TILs | None | 1.00 | 1.00 | 1.00 |
| 2. None | Irr. WM-Tr. cells | 0.71 ± 0.07 | NT | 1.16 ± 0.14 |
| 3. None | Irr. HPAF | 0.73 ± 0.09 | NT | NT |
| 4. WM-TILs | Irr. WM-Tr. cells | 1.35 ± 0.08 | 0.72 ± 0.02 | 1.14 ± 0.11 |
| 5. WM-TILs | Irr. HPAF | 0.90 ± 0.14 | 0.63 ± 0.02 | NT |
| 6. WM-TILs | IL-2 | 18.59 ± 1.90 | 11.83 ± 0.52 | 33.89 ± 1.89 |
| 7. WM-TILs | Irr. WM-Tr. cells + IL-2 | 26.39 ± 1.19[b] | 17.84 ± 0.24[c] | 42.34 ± 1.26[d] |
| 8. WM-TILs | Irr. HPAF + IL-2 | 18.40 ± 1.00 | 12.34 ± 0.67 | NT |
| B. | | | | |
| 1. PBL-T cells | None | 1.00 | 1.00 | |
| 2. None | Irr. WM-Tr. cells | 0.91 ± 0.08 | 0.82 ± 0.02 | |
| 3. None | Irr. HPAF | 0.72 ± 0.01 | 0.93 ± 0.01 | |
| 4. PBL-T cells | Irr. WM-Tr. cells | 1.21 ± 0.04 | 1.13 ± 0.01 | |
| 5. PBL-T cells | Irr. HPAF | 1.43 ± 0.04 | 2.24 ± 0.34 | |
| 6. PBL-T cells | IL-2 | 13.42 ± 0.62 | 17.62 ± 0.48 | |
| 7. PBL-T cells | Irr. WM-Tr. cells + IL-2 | 27.22 ± 0.86[e] | 30.20 ± 1.81[f] | |
| 8. PBL-T cells | Irr. HPAF + IL-2 | 12.84 ± 0.58 | 18.30 ± 1.16 | |

[a]WM tumour-infiltrating lymphocyte (TIL) line and tumour-specific T-cell line were established by repeatedly stimulating with autologous irradiated (Irr.) mucin-expressing pancreatic tumour (tr.) cells (WM-tumour cells) and 5 U $ml^{-1}$ interleukin-2 (IL-2). The proliferative response ([$^3$H]thymidine incorporation) of resting tumour-infiltrating lymphocyte line was determined in triplicates following three day incubation with mucin-expressing autologous (WM) or heterologous (HPAF) pancreatic tumour cells in the presence or absence of 1 U $ml^{-1}$ IL-2. [b–f]Significantly different from respective TILs or PBL-T-cells incubated with IL-2, [b]$P < 0.05$; [c]$P < 0.005$; [d]$P < 0.05$; [e]$P < 0.001$; [f]$P < 0.01$. NT, not tested.

**Table 5** Specific stimulatory effect of autologous tumour cells and IL-2 on tumour-infiltrating T-cell line[a]

| Responder | Stimulator | Proliferative index Expt. 1 | Expt. 2 |
|---|---|---|---|
| 1. WM-TILs | None | 1.00 | 1.00 |
| 2. None | Irr. WM-tumour cells | 0.83 ± 0.10 | 0.61 ± 0.07 |
| 3. WM-TILs | Irr. WM-tumour cells | 0.91 ± 0.05 | 1.02 ± 0.08 |
| 4. WM-TILs | IL-2 + Control Ab | 10.78 ± 0.62 | 19.22 ± 0.52 |
| 5. WM-TILs | IL-2 + L243 Ab | 9.70 ± 1.06 | 17.63 ± 0.52 |
| 6. WM-TILs | Irr. WM-tumour cells + IL-2 + Control Ab | 20.21 ± 1.67 | 31.32 ± 1.95 |
| 7. WM-TILs | Irr. WM-tumour cells + IL-2 + L243 Ab | 13.51 ± 0.63[b] | 23.42 ± 0.62[c] |
| 8. WM-TILs | Irr. Autologous EBV-B Cells + IL-2 | 12.20 ± 0.72 | 20.32 ± 0.80 |
| 9. WM-TILs | IL-2 + SP-1 Sup | 11.23 ± 0.49 | 18.91 ± 0.95 |
| 10. WM-TILs | IL-2 + DU-PAN-2 Ab | 10.73 ± 0.95 | 18.63 ± 1.11 |
| 11. WM-TILs | Irr. WM-tumour cells + IL-2 + SP-1 Sup | 22.39 ± 2.34 | 33.52 ± 2.12 |
| 12. WM-TILs | Irr. WM-tumour cells + IL-2 + DU-PAN-2 Ab | 23.52 ± 1.55 | 32.84 ± 1.74 |

[a]WM tumour-infiltrating lymphocyte (TIL) line was established by repeatedly stimulating with autologous irradiated (Irr.) mucin-expressing pancreatic tumour cells (WM-tumour cells) and 5 U $ml^{-1}$ interleukin-2 (IL-2). The proliferative response ([$^3$H]-thymidine incorporation) of resting tumour-infiltrating lymphocyte line was determined in triplicates following 3-day incubation with mucin-expressing autologous (WM) pancreatic tumour cells (passage 1) in the presence or absence of 1 U $ml^{-1}$ IL-2. For blocking experiments, irradiated tumour cells were preincubated with 20 μg $ml^{-1}$ control $IgG_{2a}$, SP-1 supernatant (Sup), L243 ($IgG_{2a}$) or DU-PAN-2 (IgM) antibody (Ab) for 60 min at 37°C and were washed in RPMI medium before adding to the responder T-cells. [b]Statistically not significant from TILs incubated with irradiated WM-tumour cells, IL-2 and control antibody; [c]Significantly different from TILs incubated with irradiated WM-tumour cells, IL-2 and control antibody ($P < 0.05$).

expected, both WM- and RC-LAK cells lysed the NK cell target, K-562 (50% and 70% respectively). The results suggest that PBMC-derived LAK cells from cancer patients did not lyse pancreatic tumour cells in the context of mucin.

### Purified pancreatic tumour cell-mucin does not elicit mucin- or tumour cell-specific T-cell response

Previous investigators (Barnd et al, 1989) observed that purified pancreatic tumour mucin directly stimulated T-cells established against mucin-expressing heterologous pancreatic tumour cells. To determine whether mucin could elicit a tumour-specific T-cell response, purified mucin from the same source used by Barnd et al (1989) was obtained and tested as described in Materials and Methods. All cells could be cultured with purified mucin and IL-2 for about 2–3 months without much expansion. As shown in Table 7, the established cytotoxic T-cell line (TP) from lymph node of a patient against irradiated T3M4 in the presence of 5 U $ml^{-1}$ IL-2, effectively lysed the stimulator cells T3M4 (78% lysis at effector:target ratio of 25:1) and at very low levels the K-562 cells (4.91% lysis). When these T-cells were cultured with 1900 U $ml^{-1}$ mucin and 5 U $ml^{-1}$ IL-2 for 5 weeks, they insignificantly killed

*© 2000 Cancer Research Campaign* 

**Table 6** Lymphokine-activated killer (LAK) cells do not recognize pancreatic tumour cells in the context of mucin

| Effector cells[a] | Target tumour cells | % Cytotoxicity Expt. 1 | % Cytotoxicity Expt. 2 |
|---|---|---|---|
| 1. WM-LAK cells | WM (autologous, mucinous) | 2.07 ± 1.19 | –0.91 ± 0.29 |
| | HPAF (heterologous, mucinous) | 49.49 ± 2.84 | 25.79 ± 0.54 |
| | K-562 (LAK-cell target, non-mucinous) | 50.83 ± 0.54 | 25.86 ± 1.35 |
| 2. RC-LAK cells | WM (heterologous, mucinous) | 26.98 ± 0.51 | 14.00 ± 1.73 |
| | HPAF (heterologous, mucinous) | 54.98 ± 1.42 | 53.05 ± 2.41 |
| | K-562 (LAK-cell target, non-mucinous) | 70.68 ± 2.69 | 61.36 ± 1.78 |

[a]Effector LAK cells (WM and RC) were obtained by culturing peripheral blood mononuclear cells from mucin-expressing pancreatic adenocarcinoma patients with 1000 U ml$^{-1}$ of human recombinant interleukin-2 for 5 days and the cytolytic activity was determined using various effector (E) to target (T) ratios. The values represent percentage specific lysis obtained at E:T 25:1 in a 4 h Chromium-51 release assay. HLA pattern of effector and target cells are given in Table 2

**Table 7** Lack of mucin-specific cytotoxic response of pancreatic cancer patients' heterologous tumour-specific T-cell or PBMCs cultured with mucin plus IL-2

| | Responder | Stimulus | Targets | % Cytotoxicity[d] |
|---|---|---|---|---|
| A. | TP-T-cells[a] | Irr. T3M4 + IL-2 | T3M4 | 78.17 ± 0.08 |
| | | | K-562 | 4.91 ± 0.84 |
| | TP-T cells generated against T3M4[b] | Mucin + IL-2 | T3M4 | 5.75 ± 0.38 |
| | | | K-562 | 4.17 ± 0.19 |
| B. | PBMCs[c] | IL-2 | HPAF | 1.38 ± 0.25 |
| | | | PANC-1 | 8.62 ± 1.20 |
| | | | K-562 | 7.79 ± 0.63 |
| | | Mucin + IL-2 | HPAF | 1.03 ± 0.38 |
| | | | PANC-1 | 10.27 ± 0.34 |
| | | | K-562 | 11.04 ± 0.08 |

[a]TP-T-cells were established from tumour-draining lymph nodes of a pancreatic adenocarcinoma patient against heterologous pancreatic tumour cells T3M4 by repeated stimulation with irradiated tumour cells in the presence of 5 U ml$^{-1}$ interleukin-2 for 2 months. [b]T3M4-specific heterologous cytotoxic T-cells were repeatedly cultured with 1900 U ml$^{-1}$ mucin and 5 U ml$^{-1}$ IL-2 for 5 weeks. [c]Peripheral blood mononuclear cells (PBMCs) from a cancer patient were repeatedly cultured with 5 U ml$^{-1}$ IL-2 or 1900 U ml$^{-1}$ mucin plus 5 U ml$^{-1}$ IL-2 for 5 weeks. The data represent the cytolytic activity of T-cell lines assessed after continuous culture against Chromium-51-labelled tumour cell targets using various effector (E) to target (T) ratios. [d]Values represent percentage specific lysis obtained at E:T 25:1 in a 4 h Chromium-51 release assay. Each experiment was repeated three times, and data from one representative experiment are shown.

**Table 8** Lack of stimulatory effect of mucin on pancreatic cancer patient's PBMCs

| Experimental groups | Proliferative index Expt. 1 | Proliferative index Expt. 2 |
|---|---|---|
| 1. PBMCs | 1.00 | 1.00 |
| 2. PBMCs + IL-2 (5 U ml$^{-1}$) | 37.13 ± 0.88 | 24.38 ± 0.94 |
| 3. PBMCs + Mucin (475 U ml$^{-1}$) | 0.56 ± 0.09 | 0.62 ± 0.01 |
| 4. PBMCs + Mucin (950 U ml$^{-1}$) | 0.37 ± 0.03 | 0.59 ± 0.07 |
| 5. PBMCs + Mucin (1900 U ml$^{-1}$) | 0.46 ± 0.13 | 0.54 ± 0.11 |
| 6. PBMCs + Mucin (2375 U ml$^{-1}$) | 0.49 ± 0.07 | 0.57 ± 0.05 |
| 7. PBMCs + IL-2 (5 U ml$^{-1}$) + Mucin (475 U ml$^{-1}$) | 34.45 ± 0.29 | 23.45 ± 0.61 |
| 8. PBMCs + IL-2 (5 U ml$^{-1}$) + Mucin (950 U ml$^{-1}$) | 34.80 ± 0.60 | 23.12 ± 0.86 |
| 9. PBMCs + IL-2 (5 U ml$^{-1}$) + Mucin (1900 U ml$^{-1}$) | 31.77 ± 1.59[a] | 21.67 ± 1.35[b] |
| 10. PBMCs + IL-2 (5 U ml$^{-1}$) + Mucin (2375 U ml$^{-1}$) | 28.93 ± 1.12[c] | 19.57 ± 1.14[d] |

The proliferative response ([$^3$H]-thymidine incorporation) of PBMCs from cancer patients was determined following 3-day incubation in triplicates with 5 U ml$^{-1}$ IL-2 or varying concentrations (475 U ml$^{-1}$ to 2375 U ml$^{-1}$) of mucin in the presence or absence of 5 U ml$^{-1}$ IL-2. [a,c,d]Significantly different from PBMCs incubated with IL-2, [a]$P < 0.05$; [b]Not significant; [c]$P < 0.01$; [d]$P < 0.05$.

*© 2000 Cancer Research Campaign*

the initial stimulator T3M4. Similarly, PANC-1-reactive CTLs, after culturing repeatedly with mucin plus IL-2 for 5 weeks, displayed an unresponsiveness to PANC-1 cells (data not shown). PBMCs derived from a cancer patient, cultured either with 5 U $ml^{-1}$ IL-2 or 1900 U $ml^{-1}$ mucin plus 5 U $ml^{-1}$ IL-2, exhibited low level killing of non-mucinous PANC-1 cells and NK cell target, K-562 cells, with negligible cytotoxicity against mucinous HPAF cells (Table 7). Culturing fresh PBMCs from cancer patients for 3 days with varying concentration of mucin in the presence of 5 U $ml^{-1}$ IL-2, did not stimulate a proliferative response over IL-2 alone (Table 8). Rather, 1900 and 2375 U $ml^{-1}$ concentrations of mucin decreased the proliferative response induced by IL-2 (11–14% and 20–22% decrease respectively; $P < 0.05$). The results of these experiments reveal that the purified mucin, (a) rendered the heterologous pancreatic tumour cell-reactive T-cells unresponsive to its target even in the presence of IL-2, and (b) did not produce mucin- or tumour-reactive T-cells from patients' peripheral blood cells even in the presence of antigen-presenting cells and IL-2. These results fail to support the contention that mucin is an antigen recognized by T-cells from cancer patients.

### DISCUSSION

Barnd et al (1989) claimed a general MHC-unrestricted recognition of mucin by heterologous T-cells based on one patient. Since an autologous cell system was not available, Barnd et al (1989) used a heterologous cell system. The results of our investigation do not support MHC-unrestricted recognition of pancreatic tumour mucin by pancreatic tumour cell-reactive heterologous cytotoxic T-cells. WM-cytotoxic T-cells established against heterologous tumour cells HPAF lysed the stimulating target cells HPAF as well as mucin-non-expressing PBL-blast cells that possessed matching MHC class I alleles with the stimulator (HPAF). These T-cells, however, did not lyse autologous tumour cells in spite of expressing mucin on the surface, further indicating an allogeneic response. Furthermore, the autologous T-cells, proliferated against their own tumour cell target WM, did not respond to heterologous, mucinous-tumour cell line HPAF, thus revealing no MHC-unrestricted recognition of mucin. Even, PBMC-derived LAK cells did not universally recognize pancreatic tumour cells that expressed mucin. If mucin were recognized, LAK cells would have killed the autologous pancreatic tumour cells that expressed mucin. In another paper, Jerome et al (1991) reported that lymph node cells obtained from two patients were alternatively stimulated by varying the stimulator cells (lymph node cells from each patient were stimulated with five different heterologous, HLA-unmatched tumour cells in an alternating fashion). It is unclear what kinds of T-cells were generated in such a situation. Although these investigators justified the use of such a method of stimulation, their observation does not unequivocally disregard the presence of alloreactivity. If one were to agree that such methodology is appropriate, it should have been employed in other studies as well. That does not seem to be the case (Barnd et al, 1989; Margarian-Blander et al, 1998).

In the present study, we did not observe a promiscuous reactivity of heterologous T-cells against a variety of mucin-expressing pancreatic and breast tumour cell lines. An initial study by Barnd et al (1989) on heterologous pancreatic tumour cell-reactive T-cells did not provide information regarding the similarities and the differences in HLA class I molecules of cells used in their experiments. Although, in a later study (Jerome et al, 1991), HLA-defined breast and pancreatic tumour cell-reactive T-cells were used in some cases, the complexity involved in the stimulation of lymph node cells with varying heterologous tumour cells by rotation precludes observation of the hidden MHC-restricted recognition. The potential alloreactivity in the heterologous tumour cell-reactive T-cell system might have contributed to the cellular immune response which the previous investigators attributed solely to MUC-1 reactivity (Barnd et al, 1989; Jerome et al, 1991; Magarian-Blander et al, 1998). Notably, one of the synthetic nine-amino acid long peptides designed from the section of the tandem repeats of mucin was shown to bind HLA-A11 and to generate peptide-specific CTLs from PBLs of several healthy HLA-A11 donors (Domenech et al, 1995). The characteristics of this HLA-restricted epitope in the T-cell response to autologous, and/or heterologous, mucin-expressing pancreatic tumour cells were not determined. Subsequent indirect studies which extended the atypical recognition of mucin by tumour cell-reactive heterologous T-cells are inconclusive (Domenech et al, 1995; Henderson et al, 1996; Magarian-Blander et al, 1996, 1998; Bohm et al, 1997; Goydos et al, 1997). Mucin has yet to be demonstrated as an antigen recognized by pancreatic tumour cell-reactive autologous T-cells.

Consistent with the present findings, other investigators (Katano et al, 1993; Wolfel et al, 1993; Peiper et al, 1997*a*) established pancreatic tumour-reactive autologous T-cell systems and demonstrated that CTLs indeed lysed autologous tumour cells in an HLA class I-restricted fashion. No significant cytotoxicity was found against autologous fibroblasts, several heterologous pancreatic cancer cell lines or an NK cell target, K-562 cells. Subsequently, Peiper et al (1997*b*) demonstrated that autologous pancreatic tumour-reactive CTLs recognized HER2/neu, a transmembrane protein with extensive homology to the epidermal growth factor receptor. These CTLs from pancreatic tumour-associated lymphocytes were shown to recognize autologous and heterologous $HER2/neu^{+}$ tumour cells in an HLA-A2-restricted fashion. In an indirect approach, other investigators detected primed MHC-restricted T-cell immunity to p21ras protein and/or peptides in some patient with pancreatic and colon cancer (Qin et al, 1995; Gjertsen et al, 1996). In the present work, autologous pancreatic tumour cell-reactive $CD4^{+}$ T-cells but not $CD8^{+}$ T-cells were generated from tumour-infiltrating and peripheral blood lymphocytes. Of note, during an initial expansion of autologous tumour cell-reactive T-cells from peripheral blood mononuclear cells, cytotoxic $CD8^{+}$ T-cells were seen (RS Selvan, unpublished observation). After a few rounds of stimulation with irradiated autologous tumour cells and IL-2, only non-cytotoxic $CD4^{+}$ T-cells were expanded. The functional role of such T-cells is currently unknown. In a melanoma system, investigators have demonstrated that autologous melanoma-reactive $CD4^{+}$ T cells possessed suppressor function against cytotoxic T-cell function (Chakraborty et al, 1990). The existence of suppressor T-cell function attributable to our autologous pancreatic tumour-reactive $CD4^{+}$ T-cells remains to be determined.

The results of the present study also raise the question whether mucin is significantly immunogenic. We did not observe stimulatory effects of purified tumour-mucin and IL-2 on established, heterologous pancreatic tumour cell-reactive T-cells. This suggests that (a) purified mucin does not replace the stimulating mucinous-tumour cells, and (b) the lack of periodic stimulation diminishes the reactivity of established T-cells against the stimulating target

© 2000 Cancer Research Campaign cells. Furthermore, we could not establish mucin- or mucinous tumour cell-reactive T-cells from patients' PBMCs with purified mucin and IL-2, even in the presence of antigen-presenting cells. Our results suggest that in our autologous system, mucin is more likely not the recognition antigen. If it were the antigen, we would have generated mucin or tumour-specific T-cell response from PBMCs, lymph node cells or splenic cells of mucinous-tumour patients, cultured with purified mucin and IL-2. We did not detect such a response in our cultures. We cannot rule out the possibility that tumour-reactive autologous T-cells might still be recognizing unique determinant(s) of mucin on tumour cells. In order to establish whether mucin on tumour cells is recognized by T cells, unique determinants need to be identified using a direct approach, such as gene transfection with cDNA library of mucinous tumour cells, and subsequent screening of transfectants with tumour cell-reactive autologous T-cell clone (Deplean et al, 1997). Interestingly, previous investigators (Barnd et al, 1989) observed that purified tumour-mucin directly stimulated the T-cells that were established against mucin-expressing heterologous pancreatic tumour cells. Further investigations explored the role of mucin as a possible tumour vaccine (Goydos et al, 1997). A recent study (McKolanis et al, 1996) noted that mucin-specific T-cells are very rare in tumour draining lymph nodes. MUC-1 has also been shown not to induce apoptosis in T-cells but to inhibit human T-cell proliferative responses (Agrawal et al, 1998; Boussiotis et al, 1998). In addition, a previous study (Agrawal et al, 1998) demonstrated that polyclonal proliferative response of T-cells was restored when IL-2 was included with purified mucin. Contrary to this observation, Paul et al (1999) found no evidence for an inhibitory role for MUC-1. Furthermore, in sharp contrast to the present study, previous investigators (Wahab and Metzgar, 1991) noted that T-cells were inhibited by HPAF cells which expressed high levels of mucin. This observation questions the immunogenicity of pancreatic tumour mucin, MUC-1.

Investigations on autologous pancreatic tumour cell-reactive T cells as well as HLA-defined heterologous pancreatic tumour cell-reactive T-cells underscore the importance of establishing several pancreatic tumour cell-reactive, autologous T-cell systems in vitro for use in the identification of novel tumour-associated antigens. For example, the identification of immunological and genetic reagents displaying the appropriate specificity will help in distinguishing between pancreatic adenocarcinoma and normal pancreas (Grem, 1997). A large number of studies have identified several novel tumour-associated antigens using autologous T-cells in other solid malignancies such as melanoma (Robbins and Kawakami, 1996). Ultimately, there is a need for systematic identification and characterization of pancreatic tumour-associated antigens. The immune responses directed against them will advance our understanding of pancreatic tumour-specific immunity and facilitate the developments of novel therapeutics, possibly including tumour vaccines.

### ACKNOWLEDGEMENTS

Special thanks to Dr Richard S Metzgar (Emeritus Professor) for his interest, encouragement and support with this work. Thanks to Kathryn M Roberson for her advice with primary culture of tumour cells; Michael S Lan (currently at the Department of Pediatrics, Lousiana State University, New Orleans, LA) for purified pancreatic tumour mucin; Frank L Tuck for assistance with immunocytochemistry; Angelica DeOliveria for devoting her time in HLA typing of tumour cells and peripheral blood lymphocytes; and Michael J Cook for flow cytometry analysis. George Padilla, Mepur Ravindranath, Rob Stephenson, Penny Miron and Scott Pruitt are gratefully acknowledged for critical comments on the manuscript. Clinical samples used in this work were obtained in accordance with the institutional review board (IRB) protocols and patient's consent.

### REFERENCES

Agrawal B, Krantz MJ, Reddish MA and Longenecker B (1998) Cancer-associated MUC1 mucin inhibits human T cell proliferation, which is reversible by IL-2. *Nat Med* **4:** 43–49

Barnd DL, Lan MS, Metzgar RS and Finn OJ (1989) Specific, major histocompatibility complex-unrestricted recognition of tumour-associated mucins by cytotoxic T cells. *Proc Natl Acad Sci USA* **86**: 7159–7163

Belldegrun A, Kasid A, Uppenkamp M, Topalian SL and Rosenberg SA (1989) Human tumor-infiltrating lymphocytes: analysis of lymphokine mRNA expression and relevance to cancer immunotherapy. *J Immunol* **142**: 4520–4526

Bohm CM, Mulder MC, Zennadi R, Notter M, Schmitt-Graft A, Finn OJ, Taylor Papadimitriou J, Stein H, Clausen H, Riecken EO and Hanski C (1997) Carbohydrate recognition on MUC1-expressing targets enhances cytotoxicity of a T cell subpopulation. *Scand J Immunol* **46**: 27–34

Borowitz MJ, Tuck FL, Sindelar WF, Fernsten PD and Metzgar RS (1984) Monoclonal antibodies against human pancreatic adenocarcinoma: distribution of DU-PAN-2 antigen on glandular epithelia and adenocarcinoma. *J Natl Cancer Inst* **72**: 999–1005

Boussiotis VA, Freeman GJ, Gribben JG, Hayes DF and Nadler LM (1998) No evidence for MUC1-induced apoptosis. *Nat Med* **4**: 1093

Chakraborty NG, Twardzik DR, Sivanandham M, Ergin MT, Hellstrom KE and Mukherji B (1990) Autologous melanoma-induced activation of regulatory T cells that suppress cytotoxic response. *J Immunol* **145**: 2359–2364

De Plean E, Lurquin C, Lethe B, van der Bruggen P, Brichard V, Renauld JC, Coulie P, Van Pel A and Boon T (1997) Identification of genes coding for tumor antigens recognized by cytolytic T lymphocytes. *Methods* **12**: 125–142

Domenech N, Henderson RA and Finn OJ (1995) Identification of an HLA-A11-restricted epitope from the tandem repeat domain of the epithelial tumor antigen mucin. *J Immunol* **155**: 4766–4774

Gjertsen MK, Saeterdal I, Thorsby E and Gaudernack G (1996) Characterization of immune responses in pancreatic carcinoma patients after mutant p21 ras peptide vaccination. *Br J Cancer* **74**: 1828–1833

Goydos JS, Elder E, Whiteside TL, Finn OJ and Lotze MT (1997) A phase I trial of a synthetic mucin peptide vaccine. Induction of specific immune reactivity in patients with adenocarcinoma. *J Surg Res* **63**: 298–304

Grem J (1997) The prognostic importance of tumor markers in adenocarcinoma of the gastrointestinal tract. *Curr Opin Oncol* **9**: 380–387

Grimm EA, Mazumder A, Zhang HZ and Rosenberg SA (1982) Lymphokine-activated killer cell phenomenon. Lysis of natural killer-resistant fresh solid tumor cells by interleukin 2-activated autologous human peripheral blood lymphocytes. *J Exp Med* **155**: 1823–1841

Henderson RA, Nimgaonkar MT, Watkins SC, Robbins PF, Ball ED and Finn OJ (1996) Human dendritic cells genetically engineered to express high levels of the human epithelial tumor antigen mucin (MUC-1). *Cancer Res* **56**: 3763–3770

Jerome KR, Barnd DL, Bendt KM, Boyer CM, Taylor-Papadimitriou J, McKenzie IFC, Bast RC Jr and Finn OJ (1991) Cytotoxic T lymphocytes derived from patients with breast adenocarcinoma recognize an epitope present on the protein core of a mucin molecule preferentially expressed by malignant cells. *Cancer Res* **51**: 2908–2916

Katano M, Nagumo F, Kubota E, Yamamoto H, Matsuo T, Hisatsugu T and Tadano J (1993) Autologous tumor-specific cytotoxic T cell clone established from tumor-infiltrating lymphocytes (TIL) of malignant ascites in the absence of recombinant interleukin 2 (rIL-2): activation by autologous tumor cell alone. *Biother* **6**: 25–32

Lan MS, Finn OJ, Fernsten PD and Metzgar RS (1985) Isolation and properties of a human pancreatic adenocarcinoma-associated antigen, DU-PAN-2. *Cancer Res* **45**: 305–310

© 2000 Cancer Research Campaign

Lan MS, Khorrami A, Kaufman B and Metzgar RS (1987) Molecular characterization of a mucin-type antigen associated with human pancreatic cancer. The DU-PAN-2 antigen. *J Biol Chem* **262**: 12863–12870

McKolanis J, Pecher G, Lotze M and Finn OJ (1996) Mucin reactive CTL induced by in vivo immunization (Abstr). *Proc Am Assoc Cancer Res* **87**: 3144

Magarian-Blander J, Hughey RP, Kinlough C, Poland PA and Finn OJ (1996) Differential expression of MUC1 on transfected cell lines influences its recognition by MUC1 specific T cells. *Glycoconjugate J* **13**: 749–756

Magarian-Blander J, Ciborowski P, Hsia S, Watkins SC and Finn OJ (1998) Intercellular and intracellular events following the MHC-unrestricted TCR recognition of a tumor-specific peptide epitope on the epithelial antigen MUC1. *J Immunol* **160**: 3111–3120

Metzgar RS, Rodriguez N, Finn OJ, Lan MS, Daasch VN, Fernsten PD, Meyers WC, Sindelar WF, Sandler RS and Seigler HF (1984) Detection of a pancreatic cancer-associated antigen (DU-PAN-2 antigen) in serum and ascites of patients with adenocarcinoma. *Proc Natl Acad Sci USA* **81**: 5242–5246

Miller G and Lipman M (1973) Release of infectious Epstein–Barr virus by transformed marmoset leukocytes. *Proc Natl Prod USA* **70**: 190–194

Paul S, Bizouarne N, Paul A, Price MR, Hansson GC, Kieny MP and Acres RB (1999) Lack of evidence for an immunosuppressive role for MUC1. *Cancer Immunol Immunother* **48**: 22–28

Peiper M, Nagoshi M, Patel D, Fletcher JA, Geogebuure PS and Eberlein TJ (1997*a*) Human pancreatic cancer cells (MPanc-96) recognized by autologous tumor-infiltrating lymphocytes after in vitro as well as in vivo tumor expansion. *Int J Cancer* **71**: 993–999

Peiper M, Goedegebuure PS, Linehan DC, Ganguly E, Douville CC and Eberlein TJ (1997*b*) The HER2/neu-derived peptide p654–662 is a tumor-associated antigen in human pancreatic cancer recognized by cytotoxic T lymphocytes. *Eur J Immunol* **27**: 1115–1123

Poland PA, Kinlough CL, Rokaw MD, Magarian-Blander J, Finn OJ and Hughey RP (1997) Differential glycosylation of MUC1 in tumors and transfected epithelial and lymphoblastoid cell lines. *Glycoconjugate J* **14**: 89–96

Pollack MS, Heagney SD, Livingston PO and Fogh J (1981) HLA-A, B, C and DR alloantigen expression on forty-six cultured human tumor cell lines. *J Natl Cancer Inst* **66**: 1003–1012

Qin H, Chen H, Takahashi M, Disis ML, Byrd DR, McCahill L, Bertram KA, Fenton RG, Peace DJ and Cheever MA (1995) $CD4^+$ T cell immunity to mutated ras protein in pancreatic and colon cancer patients. *Cancer Res* **55**: 2984–2987

Robbins PF and Kawakami Y (1996) Human tumor antigens recognized by T cells. *Curr Opin Immunol* **8**: 628–636

Selvan RS, Nagarkatti PS and Nagarkatti M (1990) Role of IL-2, IL-4 and IL-6 in the growth and differentiation of tumor-specific $CD4^+$ T helper and $CD8^+$ T cytotoxic cells. *Int J Cancer* **45**: 1096–1104

Selvan RS, Nagarkatti PS and Nagarkatti M (1991) Characterization of T lymphocyte clones isolated from BCNU-cured LSA mice. *Int J Cell Cloning* **9**: 594–605

Slovin SF, Lackman RD, Ferrone S, Kiely PE and Mastrangelo MJ (1986) Cellular immune response to human sarcomas: cytotoxic T cell clones reactive with autologous sarcomas. I. Development, phenotype, and specificity. *J Immunol* **137**: 3042–3048

Tan MH, Nowak NJ, Ochi H, Sandberg AA, Lopez C, Pickren JW, Berjian R, Douglass HO and Ming Chu T (1986) Characterization of a new primary pancreatic tumor line. *Cancer Invest* **4**: 15–23

Wahab ZA and Metzgar RS (1991) Human cytotoxic lymphocytes reactive with pancreatic adenocarcinoma cells. *Pancreas* **6**: 307–317

Wolfel T, Herr W, Coulie P, Schmitt U, Meyer Zum Buschenfelde K-H and Knuth A (1993) Lysis of human pancreatic adenocarcinoma cells by autologous HLA-class I-restricted cytolytic T-lymphocyte (CTL) clones. *Int J Cancer* **54**: 636–644.

*© 2000 Cancer Research Campaign*
